# Emerging Two-Dimensional Materials for Electromagnetic Interference Shielding Application

**DOI:** 10.3390/ijms241512267

**Published:** 2023-07-31

**Authors:** Suman Kumari, Jasvir Dalal, Vibhor Kumar, Anand Kumar, Anil Ohlan

**Affiliations:** 1 Department of Physics, Chaudhary Ranbir Singh University, Jind 126102, India; 2 School of Engineering, Rutgers, The State University of New Jersey, Piscataway, NJ 08854, USA; 3 Department of Physics, Maharishi Dayanand University, Rohtak 124001, India

**Keywords:** EMI shielding, shielding mechanism, two-dimensional materials, graphene, MXene

## Abstract

Graphene is the first two-dimensional material that becomes the center material in various research areas of material science, chemistry, condensed matter, and engineering due to its advantageous properties, including larger specific area, lower density, outstanding electrical conductivity, and ease of processability. These properties attracted the attention of material researchers that resulted in a large number of publications on EMI shielding in a short time and play a central role in addressing the problems and challenges faced in this modern era of electronics by electromagnetic interference. After the popularity of graphene, the community of material researchers investigated other two-dimensional materials like MXenes, hexagonal boron nitride, black phosphorous, transition metal dichalcogenides, and layered double hydroxides, to additionally enhance the EMI shielding response of materials. The present article conscientiously reviews the current progress in EMI shielding materials in reference to two-dimensional materials and addresses the future challenges and research directions to achieve the goals.

## 1. Introduction

**Figure 1:**
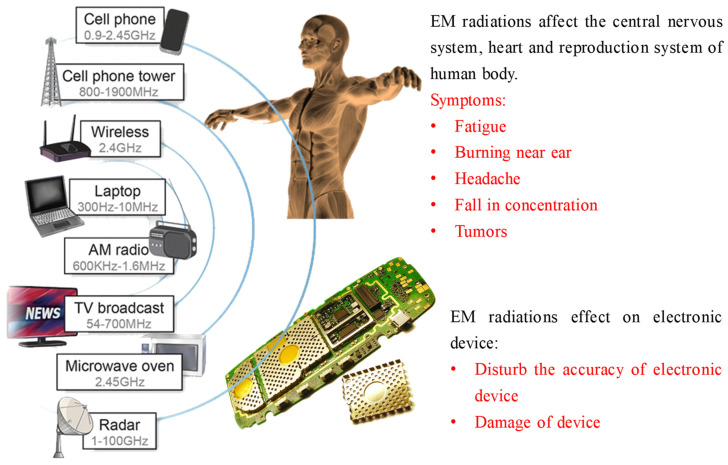
The sources of electromagnetic radiation and their effects on humans and electronics device.

In the present day, high-frequency signals are commonly utilized for operating microprocessor-controlled electronic devices, which are at the heart of the telecommunication as well as defense sectors. These electronic devices can radiate radiations out of them, i.e., electromagnetic pollution that causes malfunctioning of nearby electrical equipment. Electromagnetic interference (EMI) is a term representing such a phenomenon where one electromagnetic wave interferes with other waves, resulting in the degradation of electromagnetic signals. EMI affects the lifetime quality as well as functioning of electronic devices. Numerous reports have been published on the adverse effects of electromagnetic pollution on biological processes in wild animals [1,2,3]. There are also adverse effects such as tumors, ear problems, cancer, etc., of long-term exposure to EM radiations on human health [4]. The source and effects of electromagnetic radiation are pictorially shown in Figure 1. Due to the negative effects brought on by the widespread use of electronic devices in the surroundings, EMI has become a major source of alarm. Therefore, to regulate this problem, electromagnetic shielding must be required; the shielding mechanism protects the device from the electromagnetic radiation coming from other equipment and reduces the electromagnetic pollution by absorbing the self-generated radiation also.

The ideal material used for the shielding application should exhibit higher shielding effectiveness due to absorption along with low density, being easy to process, bulk production, low cost, being thermally stable, etc.; and possess characteristic properties like high magnetic permeability, electrical conductivity, thermal conductivity, and high aspect ratio. Therefore, there are many opportunities to produce such a material that possesses lightweight, flexible, electrical conducting, and also magnetic properties; fulfilling the needs of the modern electronics era by preventing interference from unwanted electromagnetic radiation. Stealth technology used in military equipment and fighter jets is one of the most demanding fields of highly electromagnetic radiation absorbing material. Numerous materials are being investigated for EMI shielding applications in order to meet the realistic demand for EMI shielding substances. Metals have historically been used for EMI shielding due to their great electrical conductivity. Their high density, limited mechanical flexibility, corrosiveness, and time-consuming nature, and expensive manufacturing expenses, however, limit their use. Many materials have been created and studied for EMI shielding, including metallic magnets, ferrites, ceramics, and hybrids of these materials. However, those cannot be used in the contemporary electronics era because to their high density and little flexibility.

**Figure 2:**
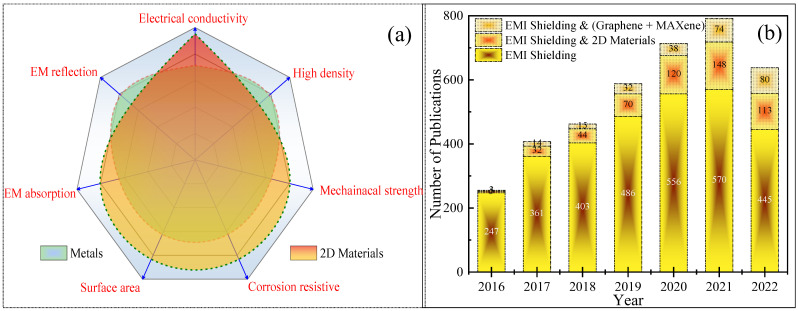
(**a**) Metals and 2D materials are compared. (**b**) Developments in EMI shielding study and the role that 2D materials have played in this area (data source: Scopus).

Due to their special qualities like strong electrical conductivity, high aspect ratio, mechanical strength, etc., several two-dimensional (2D) elements are being researched for the EMI shielding application in order to get around problems like poor mechanical strength and high density. 2D layered materials including graphene, carbon nanofibers, carbon black, carbon nanotube, transition metal dichalcogenides, 2D nitrides (boron nitride and graphitic carbon nitride), layered metal oxides, MXenes, layered double hydroxides, and black phosphorus have demonstrated tremendous shielding performance and have become the front-line materials for EMI shielding in recent years. This can be understood from the comparison between metals and 2D materials with respect to the most desirable properties for the shielding material, which has been as depicted in Figure 2a.

In recent years, research in the EMI shielding field has accelerated year by year, as observed from the hike in publications displayed in Figure 2b, indicating the importance of these shielding materials. The figure also reflects the increasing demand for 2D materials in this field, as the number of 2D-material-based shielding materials publications increased from 69 to 514 in the previous six years, because of their special characteristics. Additionally, the figure demonstrates that MXene and graphene are the most-studied 2D shielding materials in the class of 2D materials. In the existing work, we are investigating the advancements in the field of 2D materials for EMI shielding, including various types of structures, morphology, filler concentration, and tunable materials properties.

## 2. Scope of Review

Mostly, metals and their composites are enormously studied and fabricated in EMI shielding applications. Researchers are drawn to 2D composite materials with special features that have a lot of potential for EMI shielding, including such graphene, MXenes, black phosphorus, etc. These composites have many practical implementations in the fields of electrical engineering, material science, and electronic engineering, etc. These composites have an important role in the creation of highly efficient materials for EMI shielding and thermal insulation applications. As electromagnetic radiation causes hazards to human beings, EMI shielding is extremely important to escape from the harmful effects of EM radiation and to reduce the pollution caused by EM radiations to develop a sustainable environment. The purpose of this review is to know the shielding performance of various materials which helps to choose the proper material for shielding according to the strength of the electromagnetic radiation. This review provides knowledge about the radiation attenuation mechanism and the structure of the shielding materials. This review focuses on the high-performance shielding materials, keeping an eye on the environment.

## 3. EMI Shielding

**Figure 3:**
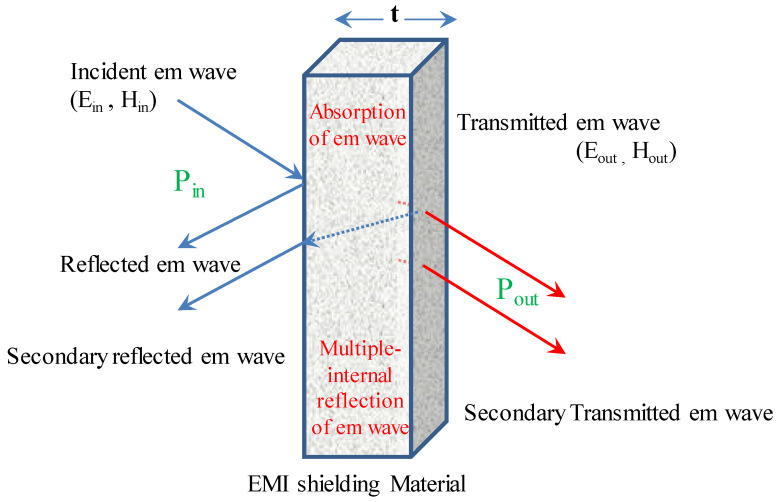
The interaction between electromagnetic waves and a shield is depicted schematically.

Through the use of a barrier made of particular materials, EMI shielding safeguards the system from electromagnetic radiation. The barrier is performing reflection, absorption, or a combined effect of both while the electromagnetic waves are incident on it. In EMI, we consider two regions of shielding: the near-field and the far-field shielding regions. It is thought to be in the far-field shielding region when the distance between the shield and the incident electromagnetic source is larger than λ/2, where λ is the wavelength of the EM wave, and in the near-field shielding region otherwise. The participation of both electric and magnetic dipoles is implemented in the near-field portions of EMI shielding, whereas the EM plane wave theory is employed in the far-field shielding zones [5]. An abstract representation of how electromagnetic waves and a shield interact is depicted in Figure 3. The incidence of electromagnetic waves on the shield causes multiple internal reflections, reflection and absorption of waves. Electromagnetic component which is neither reflected nor absorbed is residual radiation in EMI, which emerges from the shield [6].

### 3.1. Concept of Green EMI Shielding

EMI shielding can be provided through two ways: via reflection and/or absorbing the EM radiations. The shielding due to reflection has caused one big problem because, in this phenomenon, the shield also acts as a secondary source of EM radiations. Moreover, the reflected radiations can further be free to interact with electronics devices and living bodies. As results, the chance of EM interference and other damaging effects still remains alive. Thus, the shielding due to absorption phenomena is appreciated over the reflection phenomena. Concerning the facts, Cao et al. group introduced the concept of green EMI shielding index (g_s_) [7,8,9]. The term g_s_ ($=1/\left|S_{11}^{2}\right|-\left|S_{21}^{2}\right|/\left|S_{11}^{2}\right|-1$, where S_11_ and S_12_ are scattering parameters) determines the eco-friendly nature of shielding material which not only shows efficient shielding results but also controls the secondary emission of EM radiations. g_s_ > 1 represents the less secondary emission of EM radiations and significant contribution of absorption phenomena in the shielding. Foremost, Wang et al. focused on eco-friendly shielding material and synthesized the 3D eco-mimetic nanostructure of PEDOT/Fe_3_O_4_/graphene with g_s_ of 1.44 [7]. Zhang et al. prepared the WS_2_/rGO architecture that exhibits effective a green shielding effectiveness index of value > 1 [10]. 

### 3.2. Shielding Effectiveness

The ratio of the incident wave’s field strength to the transmitted wave’s field strength is known as the shielding efficacy and can be formulated as:

(1) $$\mathrm{SE}=10\log\left(P_{in}/P_{out}\right)$$

P is the strength of the plane wave; the subscript *in* indicates the incident wave and *out* indicates the transmitted wave.

A shielding material’s efficacy in blocking radiation serves as a measure of its performance (SE). Decibels are used to assess the attenuation caused by internal reflection, absorption, and other phenomena (dB). Thus, the overall shielding efficacy of a shielding substance (SE_T_) comprises SEs contributions from reflection (SE_R_), absorption (SE_A_), and multiple-reflections (SE_M_) and written as:

SE_T_ = SE_A_ + SE_R+_ SE_M_ (2)

The material’s permittivity and conductivity have a big impact on how well it shields. The conventional electromagnetic theory states that shielding efficacy for the thick shield of thickness ‘t’ having conductivity ‘σ_s_’, and magnetic permeability ‘μ’ can be given as [11].

(3) $$SE\left(dB\right)=20\frac{t}{\delta }log\;e+10log\left(\frac{\sigma_{s}}{16\omega \varepsilon_{o}\mu_{r}}\right)$$

where δ is skin depth, ω is angular frequency, $\varepsilon_{o}$ is absolute permittivity, and μ_r_ is relative magnetic permeability of shield.

The first part of Equation (3) represents shielding effectiveness due to absorption and the second part refers to reflection. Equation (3) shows that the shielding efficacy because of absorption phenomena is directly proportional to square root of conductivity, whereas reflection is directly proportional to the logarithmic value of conductivity. Therefore, shielding performance increases with an increment in the conductivity of the material. The shielding material acquiring mobile charge carriers with lower permeability yields shielding effectiveness dominated by the reflection phenomenon. The electromagnetic wave combines with the charge carrier, causing a flow of charges and creating an electromagnetic field in opposition. The external magnetic field is cancelled out by this opposing electromagnetic field. Materials with high conductivity are used for EMI shielding. With an increasing frequency of EM radiation, the reflection loss decreases.

Because the energy of the incident wave is not lost after reflection, it serves as a secondary source of EM pollution that further disrupts the other systems. Therefore, for proper shielding, EMI shielding substance must disperse the electromagnetic signals by absorbing them. To attain EM signal absorption, shielding material have to acquire electric or magnetic properties [11].

(4) $${SE}_{A}\left(dB\right)=20\;\frac{t}{\delta }\log e=20t\sqrt{\frac{\mu_{r}\omega \sigma_{s}}{2}}\log e$$

where $\sigma_{s}=\omega \varepsilon_{0}\varepsilon^{\prime \prime }$ ($\varepsilon^{\prime \prime }$ is imaginary part of complex permittivity) and skin depth (δ) $=\frac{1}{\sqrt{\pi \mu \sigma \nu }}$. The distance at which the field’s strength is reduced to 1/e of its initial value is known as the skin depth. The skin depth is an interesting phenomenon correlated with the absorption of EM waves and varies inversely with respect to the electric conductivity, frequency of electromagnetic waves and magnetic permeability of shielding substance.

Reflection occurs at different reflecting boundaries or surfaces inside the shielding material and is known as multiple reflections. Multiple reflections result in the scattering of electromagnetic waves. The multiple-reflection loss is negligible when the separation among interfaces is larger as compared to skin depth [12]. Also, SE_M_ is ignored, where absorption loss is larger than a value of 10 dB.

#### Shielding Effectiveness Calculation

The scattering parameters S_11_, S_12_, S_21_, and S_22_ measured on a vector network analyzer (VNA) are used to calculate SE. The scattering parameters signify the strength of the reflected or transmitted signal with respect to the incident signal strength. VNA can transmit or receive as well as record the EM signal intensities over a wider frequency range. Coefficients of reflection (R), transmittance (T), and the absorption (A) for the EM signal are also calculated in terms of S-parameters as follows [13]:

R = |S_11_|^2^ = |S_22_|^2^ (5)

T = |S_21_|^2^ = |S_12_|^2^ (6)

(7) A=1−T−R

Therefore, the SE for reflection and absorption in terms of S-parameters are given by:

(8) $${SE}_{R}=10\;{log}_{10}\left(\frac{1}{1-{\left[S_{11}\right]}^{2}}\right)$$

(9) $${SE}_{A}=10\;{log}_{10}\left(\frac{1-{{[S}_{11}]}^{2}}{{{[S}_{12}]}^{2}}\right)$$

Conductivity plays a crucial role in achieving higher shielding efficacy. The value of shielding efficacy required for commercial applications is 20 dB, i.e., 1% of the transmittance.

### 3.3. Shielding Measurement

Shielding tests are designed to determine the power of the transmitted wave passing through the shielding material. There are four basic methodologies that are usually used for shielding measurement [14]:

Open Field or Free Space Method;
Shielded Box Method;
Shielded Room Method;
Coaxial Transmission Line Method.

The most feasible way to assess the shielding power of an entire electronic assembly is through the open field method. The radiated emissions that escape the electronic device are measured in this method. This method does not measure the performance of the device due to the difference in the assembly of individual devices. In this test, the radiated emissions are recorded by mounting the device 30 m away from the receiver coil. Also, in this test, the conducted emissions that are transferred via the power lines are recorded.

For comparing measurements between the SE of various shielding substances, the shielded box method is used. The specimen is put down in the sample holder, which is mounted in a window in the wall of a metal box with a receiving antenna inside. A transmitting antenna that is placed outside the container measures the transmitted radiation intensities. The open port with the test specimen placed over it is used to gauge the strength of signals received at the transmitting antenna. The disadvantage of this procedure is the difficulty in obtaining electrical contact between both the test specimen and the metal container. Also, the range of frequency is also limited to about 500 MHz. To overcome the issues faced in the shielded box method, another method called the shielded room was developed. This method’s operation is essentially identical to that of the shielded box approach. To prevent EMI, the recorder, receiving and sending antennae and the signal generator are separated into separate rooms. The sample specimen must be expanded by an order of 2.5 m^2^ in size and the antennae are housed in an eco-free chamber the size of a room.

The most preferable method of EMI SE measurement is the co-axial cable transmission line method. Transmitting and receiving co-axial cables, a vector network analyzer (VNA), and a metal sample holder are used in the test setup. The losses in EM waves produced by co-axial cables are much less than antennae, and the data obtained by the co-axial cable transmission line method split into transmitted, reflected, and absorbed constituents, which is the main advantage of this method.

### 3.4. Factors Effecting EMI Shielding Performance

Foremost, there are two important factors impedance-matching (Z_r_) attenuation constant (α) that control the EMI shielding mechanism, i.e., reflection and absorption. The impedance-matching attenuation constant characteristics of the composites are governed by intrinsic characteristics, i.e., permittivity and permeability of the materials as given by Equations (10) and (11) [15]; further, the dielectric and magnetic losses are caused by both permittivity and permeability, respectively. The impedance-matching parameter control the reflection of EM radiations and flux intensity of incoming EM radiation, whereas the material’s ability to produce shielding through absorbing the radiation when EM radiations are incident and travelling through it is represented by the attenuation constant (α). According to the classical electrodynamics theory, the intensity of EM radiation decreases exponentially in the material, as $I=I_{0}e^{-\alpha x}\;(I_{0}$ is initial intensity of radiation) at the propagation distance x in the material.

(10) $$Z_{r}=\sqrt{\frac{\mu_{r}}{\varepsilon_{r}}}$$

(11) $$\alpha =\pi fc\sqrt{2\left(\mu^{\prime \prime }\varepsilon^{\prime \prime }-\mu^{\prime }\varepsilon^{\prime }+\sqrt{\left({\mu^{\prime \prime }}^{2}+{\mu^{\prime }}^{2}\right)\left({\varepsilon^{\prime }}^{2}+{\varepsilon^{\prime \prime }}^{2}\right)}\right)}$$

where ε′ and ε″ are the real and imaginary part of complex relative permittivity. μ′ and μ″ are the real and imaginary part of complex relative magnetic permeability. 

The morphology, size, and thickness of the material have an impact on EMI shielding as well. Size and morphology control the electrical conductivity and dielectric properties of the material. For the shielding material, the electrical conductivity and conducting pathways inside the material are important to obtain the higher EMI shielding. It is evident from Equation (4); the thickness of the composite is directly proportional to the shielding due to absorption. According to relation $I=I_{0}e^{-\alpha x}$, as the thickness increases, the value x increase, this assists the higher attenuation of EM radiation.

Another element that influences the efficiency of the shielding is temperature. M. Cao [16] and X. X. Wang [17] et al. reported the thermally tuned EMI shielding properties of graphene-based composite material. The authors observed that the R_L_ increased up to an optimized temperature rather than decreased, whereas EMI shielding increases with a raise in temperature. Temperature effects the charge transportation phenomena, i.e., electron hopping and migrating resulting in an increase in electrical conductivity, and, hence, conduction loss; as a result, shielding capabilities of the materials get improved. Moreover, the defects induced in the materials caused by the increase in temperature result in the attenuation of EM radiation. This is attributed to the change in structure of the material with time and temperature. 

The filler’s mass-to-volume ratio affects EMI shielding as well. The flexibility of the composites may be reduced by the presence of a higher filler concentration. The optimized filler concentration may increase polarization and, hence, dielectric loss, and it also increases the dielectric loss, as well as magnetic losses, resulting in enhanced dampening of EM radiation. 

## 4. Two-Dimensional Layered Shielding Materials

**Figure 4:**
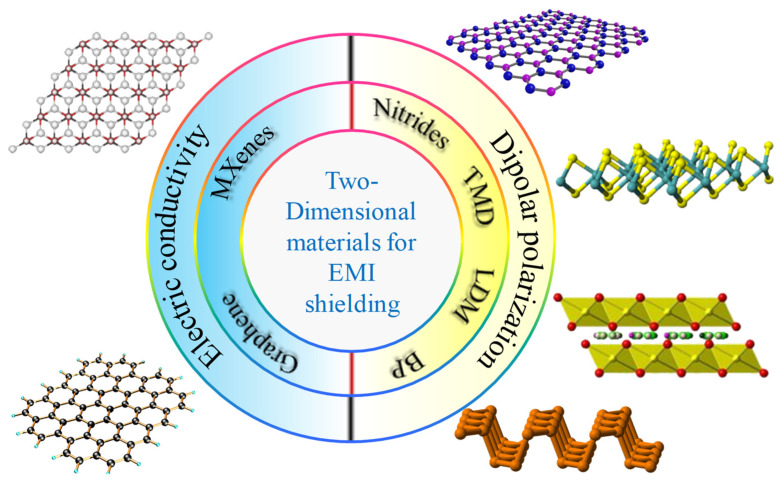
Two-dimensional materials utilized for EMI shielding purposes are shown diagrammatically.

Two-dimensional layered shielding materials such as graphene was studied because of their remarkable high surface area, tunable band gap, electrical conductivity, high charge mobility, and thermal conductivity. Such exceptional properties of graphene make it a superior material and motivate researchers to synthesize more 2D substances like carbon nanotube, transition metal dichalcogenides, hexagonal boron nitride and graphitic carbon nitride, layered metal oxides, transition metal dichalcogenides (e.g., MoS_2_, TiS_2_, TaS_2_, etc.), black phosphorus (BP), layered double hydroxides, silicene, and MXenes as shown in Figure 4. Ultrathin or 2D layered materials can meet the current requirements of the modern world’s applications. 

The EMI shielding results of 2D materials and their composites are summarized in Table 1. The most recent status of 2D materials in the realm of EMI shielding is anticipated to be provided by this review. This work discusses the shielding response of materials based on graphene, h-BN, TMDs, LDHs, BP, and MXene.

### 4.1. Graphene-Based Shielding Materials

Graphene is the most-studied material in different fields of research owing to its excellent properties like high electrical conductivity (~60 S·m^−1^), exceptional charge carrier mobility (~10,000 cm^2^V^−1^s^−1^), high surface area (~2600 m^2^g^−1^), high thermal conductivity (~5000 WmK^−1^), large aspect ratio, and excellent mechanical properties, i.e., Young’s modulus ~1 TPa [62]. In a benzene-ring/honeycomb grid configuration, a single layer of sp^2^-hybridized carbon atoms is what makes up graphene. In recent years, graphene has become the most potential candidate for EMI shielding because of its remarkable characteristics.

A. K. Geim and K. S. Novoselov synthesized the first graphene via a mechanical exfoliation technique in 2004 [63], and, later on, the synthesis of graphene has been accomplished using a wide variety of techniques. In 2009, Chen et al. [31] foremost reported the shielding result of graphene-sheet-based graphene/epoxy composites in the Ku-frequency band. The author observed that electrical conductivity increased with wt. ratio of graphene (synthesized modified Hummers method) and, in the same manner, SE also increased. The first experimental work on monolayer graphene (prepared via CVD method) employed in EMI shielding was presented by Hong et al. [21]. A monolayer of graphene has an average EMI SE of 2.27 dB, which is around seven times greater than that of gold film, and could block 40% of incident EM waves. EMI SE was increased with the number of graphene layers because conductivity also increases, which is 5.25 × 10^6^, 5.64 × 10^6^, and 5.92 × 10^6^ S·m^−1^ for mono, double, and triple layer graphene, respectively. For monolayer graphene, it was revealed that absorption phenomena contributed 90% of the total SE. However, as graphene layers are added, the relative contribution of absorption to total EMI SE decreases while the contribution of reflection increases. It indicates that the basic shielding mechanism of graphene is the same as that of Au film [64]. The calculated SE values using plane-wave theory for graphene were well-matched with the measured SE values of graphene for all thickness. It is suggested that graphene consists of infinite homogeneity and isotropy upon which plane-wave theory could be applied like in the case of metals.

**Figure 5:**
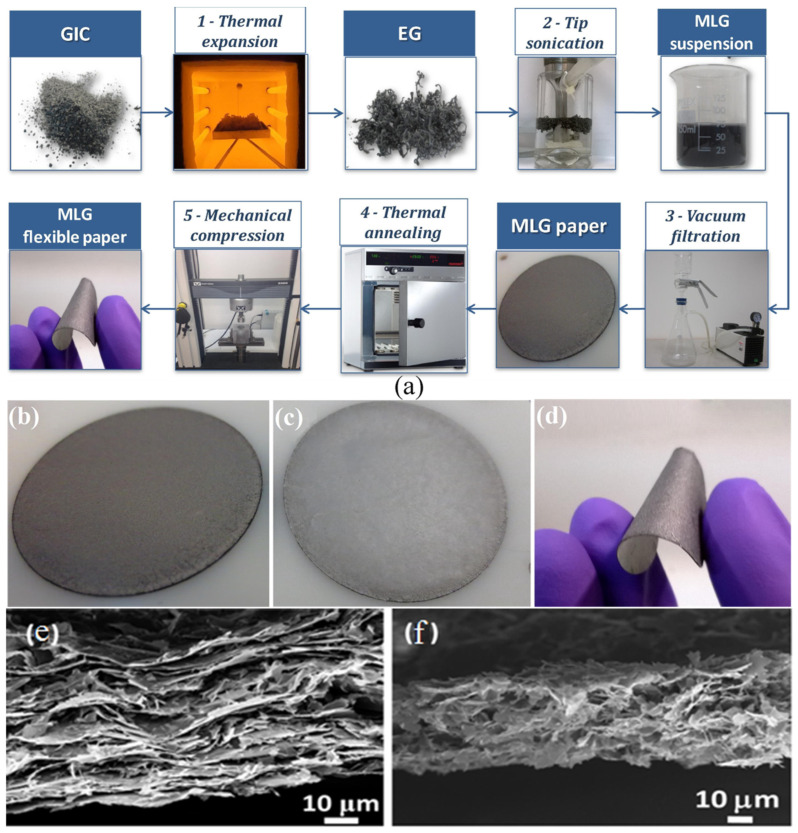
(**a**) MLG flexible paper manufacturing process schematic; MLG paper (**b**) before and (**c**) after 5 MPa mechanical compressions; and (**d**) MLG paper that has been pressed and is flexible. SEM images of MLG paper (**e**) P-DMF1, and (**f**) P-NMP1, respectively. “Reprinted from [24], Copyright (2015), with permission from Elsevier”.

Another experimental study found that five layers of graphene could absorb ~65% of the incident radiation at the frequency of 1 GHz. In addition, the stacking of six graphene layers together can provide 43 dB EMI SE [65]. Further, the shielding potential of multilayer graphene (MLG) paper produced by thermo-chemical exfoliation of graphite intercalation compounds (GICs) was also investigated [24]. It is an environmentally safe method for producing graphene paper that conducts electricity. Multi-layer graphene consists of stacked graphene sheets having a thickness range of 1 to 10 nanometers. The MLG paper was produced by first obtaining the expanded graphite (EG) from the thermal expansion of GICs. A step-by-step procedure is shown in Figure 5a. The MLG flexible paper was created by mechanically compressing the MLG paper that had been produced following vacuum filtering of the homogeneous colloidal suspension of MLGs as represented by Figure 5b–d.

The structure of produced graphene paper is highly porous, as shown in the microscopic pictures of the sample depicted in Figure 5e,f. The average contact area between parallel positioned MLG layers was reduced by higher porosity. The porosity of the paper necessitates reducing pore size in order to enhance electrical conductivity. To minimize the impact of porosity, the MLG paper generated in the single-step annealing cycle was mechanically compressed at room temperature. Compression of porous graphene (PG) films can effectively reduce the size of pores in films, thereby improving their electrical and thermal conductivities. More than 1400 S/cm of electrical conductivity was demonstrated by the MLG article, when mechanically compressed at 5 MPa. Both the thermal annealing as well as the mechanical compression affects the MLG paper’s sheet and the actual electrical resistance that is DC. The enhanced electrical conductivity leads the domination of reflection of the incident EM radiations which was 98–99% of the incident power.

**Figure 6:**
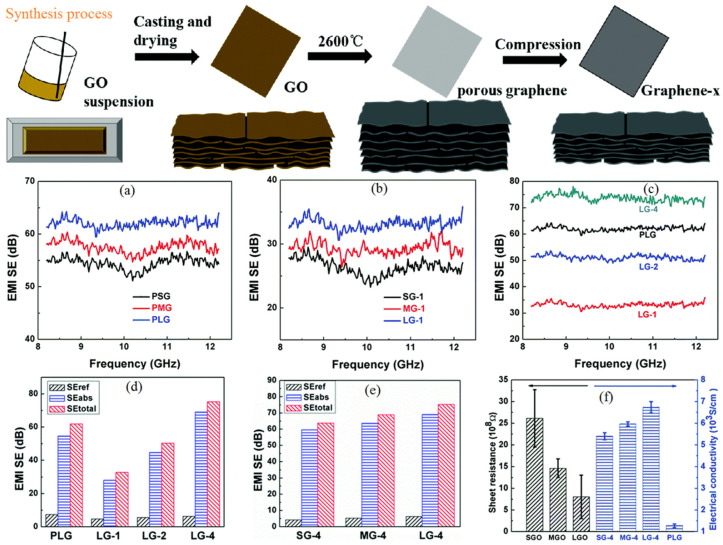
Development procedure of porous graphene sheets where x is representing number of PG sheets (tightly grouped). The EMI SEs values of (**a**) the PG sheets, (**b**) single-pour graphene sheets, and (**c**) large-sized flakes graphene films. SE_total_, SE_ref_, and SE_abs_ at approximately 10 GHz (**d**) PLG, LG-1, LG-2, and LG-4 sheets; (**e**) SG-4, MG-4, and LG-4 films; and (**f**) GO and graphene sheets’ electrical conductivity [69].

Generally, a graphene produced through the CVD technique or mechanical exfoliation is hard to process for application use. Due to the lower dispersion of as-synthesized graphene in most solvents, it is demanded for ease of processing of the composite or hybrid fabrication. Therefore, for practical applications, graphene oxide (GO) has better potency towards EM wave absorption compared to native graphene and is the most accessible precursor of graphene for making graphene films for better EMI shielding. Reducing GO films and exfoliating GO from graphite oxide are two methods that can be used to produce graphene films on a massive scale [66,67]. The oxygen-containing functional groups (such as hydroxyl, carbonyl, epoxide, and carboxyl groups) that are present on GO’s basal planes and corners are what give it its insulating qualities. However, by annealing at high temperatures, such oxygen-possessing functional groups can be quickly eliminated [68]. Shen et al. made extremely thin graphene sheets having a thickness of 8.4 µm, value of EMI SE ~20 dB, and in-plane thermal conductivity of value 1100 W·m^−1^·K^−1^, using large-area GO that was evaporated until dry, followed by thermal annealing at 2000 °C for reduction and graphitization of GO film [27]. Xi et al. produced foam-like sheets of graphene with EMI SE with in the order of 65–105 dB by raising the graphitization temperature and the reduction temperature at 3000 °C [32]. In a different study, graphene films were created using three different GO/H_2_O dispersions that contained GO flakes of various sizes (e.g., 5–8 mm, 20–30 mm, and 40–50 mm, known as SGO, MGO, and LGO), and were diluted with water to 8.0 mg/mL. The dispersions were then mechanically stirred before being bar-coated on PET plates, and graphitized at 2600 °C for 4 h. The process is schematically shown in Figure 6 [69].

Materials with higher porosity are of importance for shielding applications. The porous graphene films produced from large-sized GO flakes (PLG) show higher EMI SE value of 7.8 dB than the smaller-sized ones owing to their superior thermal as well as electrical performances as shown in Figure 6a. Two and four PLG films were compressed together to make LG-2 and LG-4, respectively. Similarly, porous graphene films produced from medium-sized GO flakes (PMG) and from small-sized GO flakes (PSG) were compressed to make MG and SG films, respectively. As shown in Figure 6c, LG-4 (14 m-thick sheet) had the highest EMI SE of all the films, measuring 73.7 dB, and the highest specific SE (SSE) divided by thickness (t), measuring 25,680 dB·cm^2^/g with the heat conductivity of 803.1 W·m^−1^·K^−1^. In addition, researchers explored the EMI shielding process in big flakes and SG-4 and MG-4 films (SE_total_, SE_abs_, and SE_ref_, at 10 GHz of the graphene). From the figure, it can also be observed that shielding is dominated by absorption phenomena instead of reflection. When LG-4 film was compressed, its electrical conductivity rose to 6740 S·cm^−1^ (Figure 6f), which significantly reduced the shield’s skin depth and enhanced SE_abs_. Lai et al. fabricated highly conductive porous graphene films using a confined foaming method [70]. First, three separate Hummers’ procedures were used to prepare the GO samples: (a) pre-oxidation modified Hummers’ method (PGO), (b) classic modified Hummers’ method (CGO), and (c) low-temperature modified Hummers’ method (LGO). The graphene oxide films were produced by drying a dispersion of graphene oxide at room temperature after it had evaporated in petri plates. The GO paper was then peeled off and sandwiched and tightly clamped between two glass plates. The desired thickness of GO films was produced by placing the spacers between the glass plates. The resulting GO films were then annealed for 2 h at 90 °C to obtain porous graphene films. PGO, CGO, and LGO each produced a porous graphene oxide film that was designated as a PPGF, CPGF, and LPGF, respectively. The LPGF film with a 200 µm thickness provided an outstanding EMI SE value of 43.8 dB in the X-band. Further to that, the PGFs possessed excellent folding resilience [70]. 

In a different study, reduction-induced foaming of GO film combined with a spatial confinement technique was used to create flexible and perforated graphene films (PGFs) [71]. The 20 µm-thick GO film, which attained an EMI SE value of 33.1 dB and 38.6 dB, was used to create the 100 µm- and 200 µm-thick PGFs. Further, it was found that annealing PGFs at 1000 ºC for 2 h in nitrogen could significantly increase their electrical conductivity and EMI SE values. Having extraordinarily high EMI SE values of 56.1 and 63.0 dB, the annealed porous graphene films of thickness 100 µm and 200 µm (APGF-100 and APGF-200, respectively) were thick enough to block and absorb 99.9998% and 99.99995% of incident radiation with just 0.0002% and 0.00005% transmission [71]. The RGO/CNF@Ag-Fe_3_O_4_ (RGCF) porous sheet was created by Guo et al. using a vacuum-assisted filtration technique and a hydrazine-induced foaming procedure [72]. RGCF films with 10, 20, and 30 mg of Fe_3_O_4_ were labeled as RGCF-1, RGCF-2, and RGCF-3, respectively. By providing a significant amount of area for multiple EM wave reflection and dispersion, the RGCF-3 film was able to obtain an EMI SE value of 21 dB [72].

**Figure 7:**
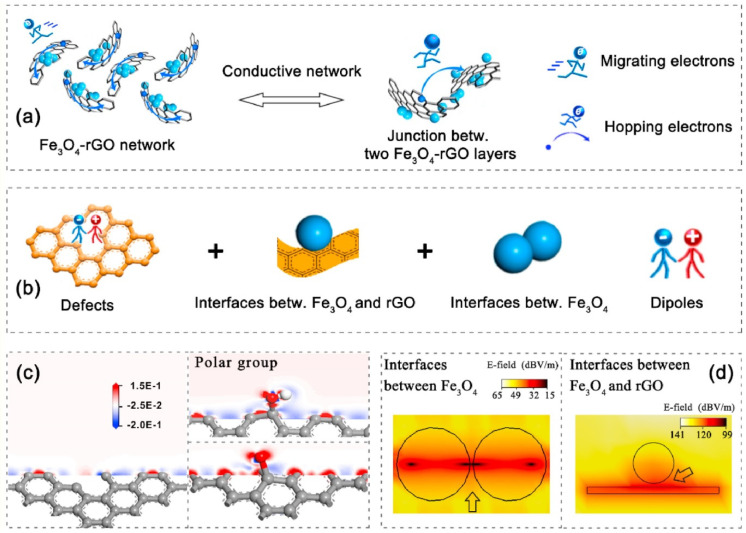
A schematic illustration of responsible phenomena for EM shielding. (**a**) The electrical conductive phenomena (via migration and hopping of electrons), (**b**) induced relaxation phenomenon (via defects, interfaces, dipoles), (**c**) distinct charge densities related to polar functional groups, and (**d**) distinct electric field intensities for various interfaces. “Reprinted from [17], Copyright (2021), with permission from Elsevier”.

The various phenomena responsible for the shielding in graphene composites have been reported. Cao et al. reported the heterostructures of graphene decorated with Fe_3_O_4_ and explore the polarization and conduction phenomena that occurred in the heterostructures that contribute in the shielding [17]. The dielectric properties are contributed by the electron, atom, and molecule polar functional group attached to graphene sheets, defects and interfaces of graphene and Fe_2_O_3_ that regulate the polarization, and conduction phenomena as shown in Figure 7. It is reported that the charge distribution around the different functional groups and defects is not symmetrical. Also, the accumulation of charges/electric field/polarization is different, related to the types of interface. It is noticed that polarization lag of a higher frequency (value of frequency depends on size of dipole and chemical bonding) results in relaxations at the cost of EM energy dissipation. Alone, the graphene sheet acts an EM radiation reflector while, after the decoration of Fe_3_O_4_, the magneto-dielectric properties synergically improve the impedance matching, resulting in higher shielding produced via the absorption phenomena [73,74].

**Figure 8:**
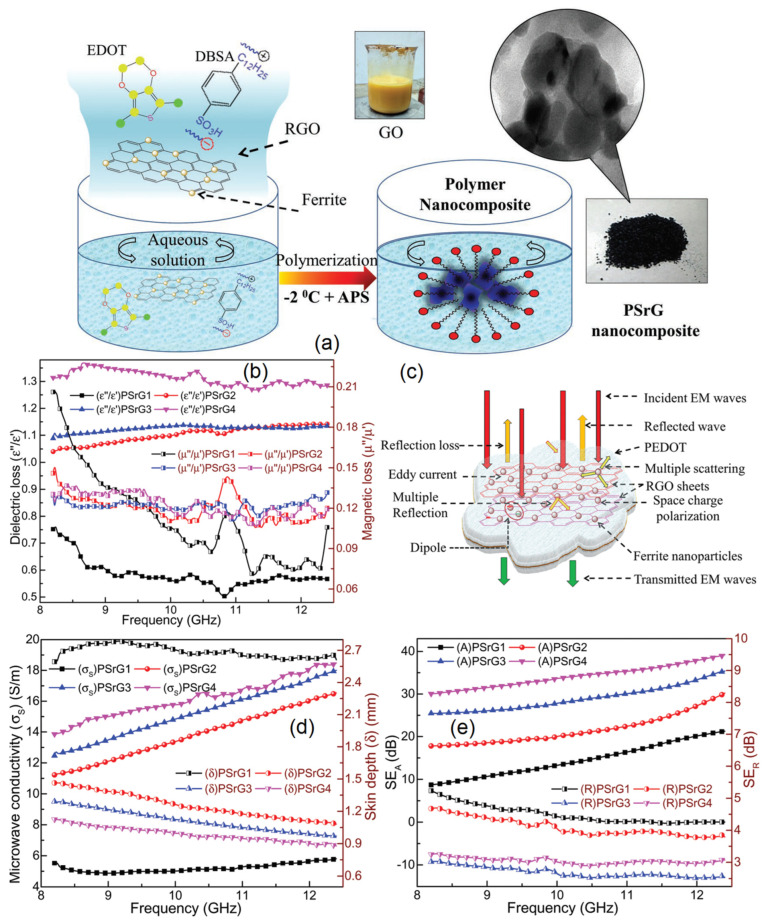
(**a**) Graphene-based polymer composite synthesis process. (**b**) Dielectric and magnetic properties; (**c**) various possible phenomena; (**d**) microwave conductivity and skin depth; and (**e**) X-band frequency response of the sample’s SE_A_ and SE_R_ [77]. Reproduced with permission [77], Copyright (2019) WILEY-VCH Verlag GmbH & Co. KGaA, Weinheim.

It is advantageous to use polymer matrices to create graphene-based polymer composites for EMI shielding implementations, because polymer is helpful in establishing an excellent conductive network of graphene sheet inside the grains that assist the shielding properties; also, due to low cost, ease of processing, good abrasion, resistance to corrosion, and ductile behavior, the graphene–polymer composites are preferred. The most important is that properties of the polymer composites can be easily changed, i.e., from dielectric to magneto-dielectric by incorporating magnetic filler [75,76]. As shown in Figure 8a, Dalal et al. described the development of core-shell-morphology-based reduced graphene oxide (RGO)/poly (3,4-ethylenedioxythiophene) (PEDOT) nanocomposites that include SrFe_12_O_19_ as magnetic filler [77]. In the PEDOT matrix, RGO and PEDOT create an electrical conducting network, and the combined dielectric-magnetic properties improve impedance matching for incident radiation, resulting in the enhanced shielding efficiency via absorption phenomena. The responsible active mechanisms (i.e., conduction loss, polarization effects, and magnetic loss that dominated by eddy current loss) for the shielding are depicted in Figure 8c. For a sample that is 2.5 mm thick, nanocomposites have a high EM SE of 42.29 dB at 12.4 GHz and exhibit attenuation of more than 99.999% attributed to enhanced electrical conducting network, polarization, and magnetic losses related to wt. ratio of magnetic filler. 

The shielding results can be supported by the analysis of dielectric and magnetic losses, i.e., the relative complex permittivity ($\varepsilon_{r}=\varepsilon^{\prime }-{j\varepsilon }^{\prime \prime }$) and relative complex permittivity ($\mu_{r}=\mu^{\prime }-{j\mu }^{\prime \prime }$). The storage capacity and energy dissipation potential of EM waves are signified by the real and imaginary components of the $\varepsilon_{r}$ and $\mu_{r}$ [78]. The dielectric properties are functions of polarization and electrical conductivity (depending on the inter-network of graphene sheets in the composite). The interfacial polarizations and space charge polarizations are the main causes of the dielectric loss in the microwave frequency [79]. The hysteresis loop, eddy current effect, and magnetic domain wall resonance in the frequency range are all responsible for the magnetic loss in the composite [80]. Dielectric and magnetic losses are depicted in Figure 8. From the figure, it is shown that, with the increase in losses, the shielding effectiveness performance increases simultaneously. In 2021, in order to create a magnetic hybrid, Ni nanoparticles were anchored to graphene. Due to the reducibility of the C atom on graphene, Ni(OH)_2_ nanoribbon and GO used as precursors both were in situ reduced at the same time during the heat reduction process. The produced film has excellent flexibility, with an EMI SE of 32.2 dB, and the obtained electrical conductivity is 262.7 S/m. This improvement is attributed to good electrical conductivity, and multi-level EM reflection from the multiple surfaces [81]. It also reported that graphene oxide can strengthen the shielding along with the mechanical properties of the material such as the interlaminar shear strength, transverse fiber bundle tensile strength, and the interfacial shear strength [82].

The methods used to produce graphene are usually top-down and bottom-up approaches which do not offer mass production of the graphene. Because carriers in graphene are of high mobility, impedance mismatches between the material and the air are produced. In order to design graphene electromagnetic shielding films, we still need to create new methods, find fresh resources, and develop the best materials. This will help us to better manage the EM wave pollution and pave the way for a better future for all of humanity.

### 4.2. D Nitrides (Boron Nitride and Graphitic Carbon Nitride)

**Figure 9:**
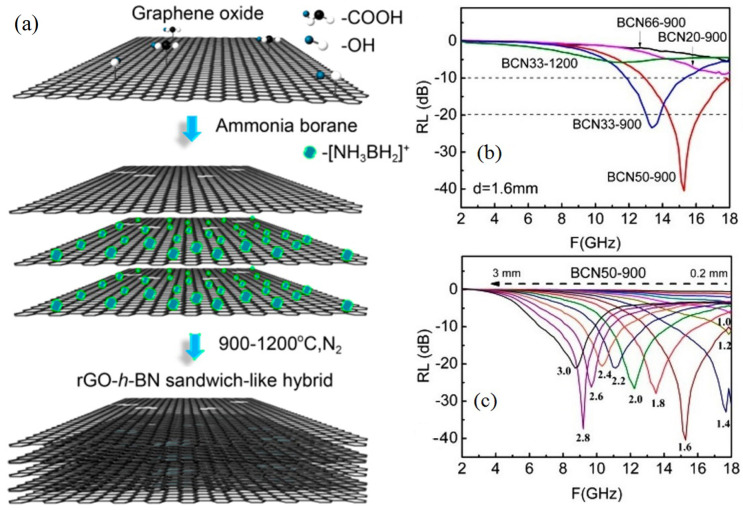
(**a**) An example of how rGO-h-BN hybrids form. Calculated R_L_ curves obtained from wax composites; (**b**) various samples at d = 1.6 mm and (**c**) BCN50-900 at different thicknesses. “Reprinted with permission from [41]. Copyright (2016) American Chemical Society”.

An isomorph of graphene, hexagonal boron nitride has properties and a crystal structure that are identical to those of graphene [83]. It is composed of nitrogen and boron atoms which are merged according to various hybridization modes to obtain a distinct phase-structured boron nitride. Boron nitride contains an infinitely extended hexagonal grid of B and N atoms arranged according to ABAB symmetry along the C-axis [84,85,86,87]. It plays the role of both substrate and capping layer, supporting intrinsic physical characteristics such quantum confinement effects. Despite being an electrical insulator and a strong heat conductor, h-BN excels as a 2D dielectric due to its level surface, absence of dangling bonds, and controllable atomic layer structure. The majority of transportable electrical gadgets like cellphones, laptops, etc. can benefit from it, that require thermal conducting material [27]. Kang et al. prepared sandwich-like hybrid structures with h-BN for EMI shielding applications and dissipated heat simultaneously. A hybrid composition of h-BN with a semi-conductive filler such as reduced graphene oxide (rGO) is reported to improve properties such as thermal conductivity, impedance matching, and, thus, shielding performance [41]. The rGO/h-BN hybrid was synthesized by heat treatment from the mixture of GO and ammonium borane; the interaction between the ammonium borane (AB) cation with the GO’s negatively charged oxygen enhances the self-assembly of the hybrids as shown in Figure 9a [37]. Different hybrid samples of rGO/h-BN were prepared by changing the ratios of the mass of raw materials and varying the annealing temperature in N_2_.

The complex permittivity and EMI shielding properties can be altered by changing the content of h-BN. For a material used for shielding applications, the value of reflection loss (R_L_) ought to be lower than 10 dB, i.e., 90% attenuation of the EM radiation [88]. With a wax composite having 1.6 mm thickness containing 25% rGO/h-BN (BNC50-900), the maximum R_L_ observed was 40.5 dB at a 15.3 GHz frequency, as shown in Figure 9b [41]. Absorption peaks change as thickness rises, moving to a lower value of frequencies. The complete reflection coefficients for BCN50-900 are shown in Figure 9c. A thickness of 0.2 to 3.0 mm is attainable. A reflection loss of less than 20 dB was attained in the frequency and thickness range of 8.6–18.0 GHz and 1.4–3.0 mm; however, at 15.3 GHz and 1.6 mm, 40.5 dB was measured.

**Figure 10:**
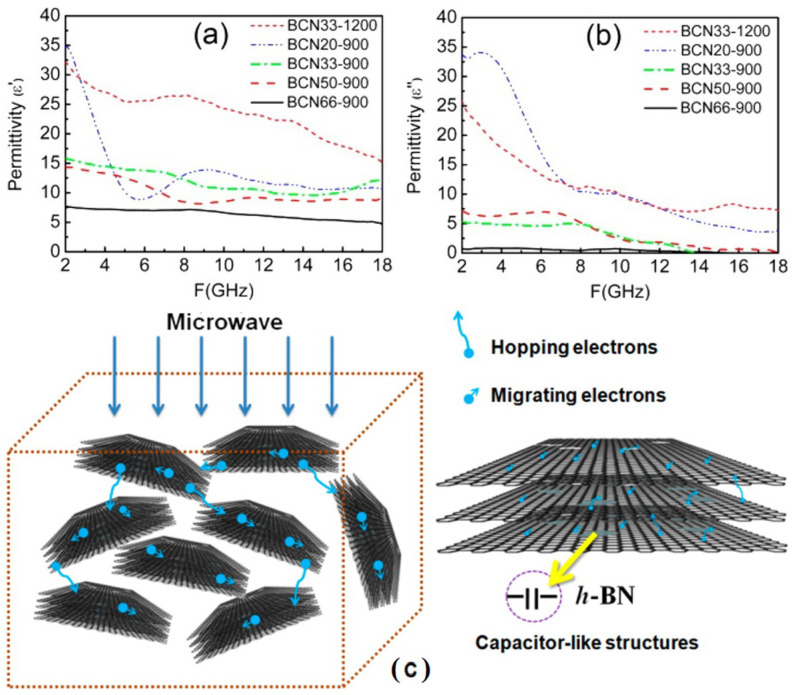
Wax composites with complex permittivity; (**a**) real part (ε′) of permittivity, (**b**) imaginary part (ε″), and (**c**) diagram showing electronic transit and microwave dissipation. “Reprinted with permission from [41]. Copyright (2016) American Chemical Society”.

The proposed mechanism of shielding performance due to absorption is shown in Figure 10. Free and newly created graphene electrons will move inside the hybrids, hop over the boundaries of graphene layers, or pass through the hybrids as microwave waves propagate through wax composites containing rGO-h-BN hybrids. As a result, these electrons contribute to the high dielectric tangent loss. The addition of h-BN to graphene causes the development of grains, which leads to the development of polarization and, ultimately, a capacitor structure on boundaries. Polarization can also be produced by the defects induced due to dipoles brought on by carbon alterations or oxygen vacancies. For microwave propagation and consumption, the outstanding impedance matching is caused by all h-BN-related flaws. In most cases, this has a big effect on the increment of ε′ rather than ε′′ (Figure 10a,b). Higher mass ratios of AB result in hybrids with higher band gaps and weaker conductivities, which is advantageous for microwave transmission in crosses. 

In addition, the rGO/h-BN hybrids’ microscale sandwich-like structure significantly increases the ways and opportunities for electron migration and hopping, enhancing the outstanding impedance matching as well as the reduction of dielectric loss. Such lightweight hybrids can be employed in the military and civil sectors where these properties are highly expected. 

A versatile film with multilayer GO/polymer and amino-functionalized h-BN/polymer was described by Zhang et al., and demonstrated superior EMI SE, great electrical insulation, and excellent thermal conductivity [38]. The author used a facile method to produce an ordered multilayer film via layer-by-layer (LbL) casting process. The multilayer film (having 11 layers and thickness of 235.2 µm) gives the EMI SE as 37.9 dB, and electrical screening (breakdown power of 1.52 M·V·m^−1^) and in-plane heat conductivity as 12.62 W·m^−1^·K^−1^, simultaneously [38]. Furthermore, Lv et al. prepared a graphene/g-C_3_N_4_ MWA hybrid absorber for microwaves. This mixture was prepared using g-C_3_N_4_ nanosheets having a composition of 15 wt% deposited on graphene, employing the liquid phase technique followed by mixing homogeneously into the wax. It was found that the composite having 1.5 mm thickness and 10 wt% ratio of filler had an R_L_ of 29.6 dB with an associated absorption bandwidth of 5 GHz in the frequency region of 12.8 GHz to 18 GHz [43]. Sandwich-like structures were created utilizing h-BN to simultaneously reduce undesired EM radiation and disperse heat. The thermal conductivity and impedance matching of the hybrid composition can be improved by combining it with a semi-conductive filler like rGO. Thus, the layered structure of h-BN is very helpful to the shielding performance through enriching the impedance-matching properties. CB/BN@Fe_3_O_4_/cellulose composite papers were fabricated with an outstanding EMI SE in the frequency ranging between 8.2–12.4 GHz. A higher EMI SE 68.2 dB was obtained with a filler of 12 wt% for the multilayer of this composite as related to its monolayer [89]. Graphite carbon nitride nanotubes/cobalt@carbon (GCNNs/Co@C) composites displaying a castor fruit structure were fabricated with minimum reflective loss of −63 dB having a 19.6 mm thickness. An extremely thin film of 1.51 mm thickness obtained actual absorption bandwidth at 4.44 GHz covering the entire X and Ku bands [90]. When bulk materials are exfoliated to monolayers, changes in the characteristics of nitride composites are produced and, therefore, affect their shielding performance. To overcome this difficulty, transition metal dichalcogenides (TMDs) are introduced.

### 4.3. Transition Metal Dichalcogenides

Two-dimensional TMD sheets have a general formula MX_2_ (as X-M-X) with an M^4+^ transition metal element (M = Mo, W, V) from the groups IV, V, and VI, and X (X = S, Se, Te) is a chalcogen [91,92]. The layered structure of graphite is inherited by many transition metal dichalcogenides (TMDs). One unit layer in TMDs has a thickness of three atoms, and it contains a transition metal (M) atom surrounded by two chacogens (X), creating the stoichiometric compound MX_2_. In contrast to the interlayer links that connect two MX_2_ slabs, which are typically van der Waals bonds, intralayer bonds are covalent. TMDs can be exfoliated down to single layers owing to poor van der Waals bonding. Among all TMDs such as MoS_2_, TiS_2_, TaS_2_, WS_2_, MoSe_2_, WSe_2_, etc., MoS_2_-based materials are most-studied for EMI shielding. Molybdenum disulfide (MoS_2_) naturally occurs as an anisotropic semiconductor molybdenite. TMDs exhibit different polymorphs and stacking polytypes, and the metal co-ordination of an individual MX_2_ layer may possess any of trigonal prismatic or octahedral geometry. MoS_2_ occurs naturally in the 2H phase and exists in three polytypes. Synthetic MoS_2_ contains a 3R phase but the metal co-ordination is trigonal prismatic in both cases [93]. TMDs materials lead to enhanced impedance matching with the free space owing to their lower conductivity and dielectric constant as compared to those of graphene. They are the excellent choices for EM wave dampening due to their qualities. First, MWA performance of TMD was described by Ning et al. based on comparison between few-layer MoS_2_ nanosheets and bulk MoS_2_ from 2 to 18 GHz frequency. A top-down exfoliation technique was used to create a sample of few-layer MoS_2_ nanosheets. With 60% wax matrix loading and MoS_2_ film composite of 2.4 mm thickness, the R_L_ is 38.42 dB, which is 4X higher than MoS_2_-bulk composite sheet, and the effective absorption bandwidth is 4.3 GHz ranging from 9.6–13.76 GHz. The polarization of defect dipoles resulting from Mo and S voids as well as dielectric relaxation events improved the MA performance of MoS_2_-NS [46]. 

**Figure 11:**
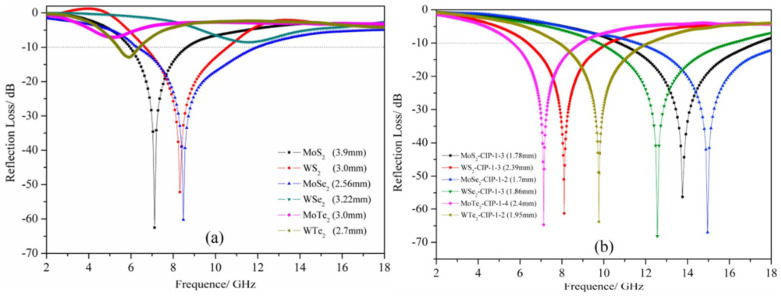
MX_2_-CIP/paraffin composite R_L_ curves (**a**) matching thicknesses in, and (**b**) in the frequency range of 2–18 GHz, with an ideal MX_2_ to CIP ratio at their respective matching thicknesses. “Reprinted from [48], Copyright (2018), with permission from Elsevier”.

In different study, Xie et al. [47] fabricated the RGO/MoS_2_ nano-sheets via an in situ hydrothermal method in which MoS_2_ layers were developed on RGO surfaces and their EM absorption characteristics were analyzed. Composite of RGO/MoS_2_ nano-sheets, with ratio filler loading of 20 wt%, showed an effectual EM absorption bandwidth of 5.7 GHz (10.72–16.44 GHz) and an R_L_ of 60 dB due to enhanced electrical conductivity, dielectric properties, and impedance-matching response. Foremost, J. Wang et al. [48] reported the shielding capability due to absorption phenomena of bulk TMDs and carbonyl iron powder (CIP) composites. MX_2_ was composited with CIP using vibrating ball milling, followed by adding the composite absorbent powder into the melted paraffin wax at 80 °C. MX_2_-CIP composites’ R_L_ curves were compared with the R_L_ curves of pure MX_2_ (80% by weight of absorbent) between frequency range from 2 to 18 GHz as shown in Figure 11a. It was discovered that MoSe_2_ had a slightly higher lower R_L_ value than MoS_2_, but that its correlating width was the thinnest of all the samples. Therefore, MoSe_2_/paraffin with 80% by weight loading exhibited best MA performance with an R_L_ of 60.23 dB at frequency 8.48 GHz, a 2.56 mm correlating thickness, and an effectual absorption bandwidth (R_L_ < 10 dB) of up to 5.68 GHz in a frequency ranging from 6.32–12.0 GHz. Further, with mass ratios ranging from 1:1 to 1:6, the MA performance of MX_2_-CIP composites was examined. The best MA performance was demonstrated by WSe_2_-CIP-1-3 (corresponding to sample having a WSe_2_:CIP ratio 1:3) with 80 weight percent filler loading as shown in Figure 11b. This composite had an R_L_ of 68.14 dB, correlating thickness of 1.86 mm, and an effectual absorption bandwidth value of 6.32 GHz [48]. High magnetic loss, enhanced impedance matching, and mild electric loss are the major characteristics. Thus, TMD-composite-based materials are favorable for efficient shielding applications. Biodegradable shielding material of MoS_2_ with high absorption and less reflection is fabricated by drenching and carbonization into cellulose paper which results in the absorption of EM up to 15 dB and EMI SE performance of 28 dB, indicating environment friendly and pollution suppressing composites [94]. Al@MoS_2_/rGO nanohybrids with micro flower-like morphology in 3D is reported which is operational in the microwave frequency range between 8–12 GHz. An EMI SE value of 33.38 dB was obtained with a doping of 12% Al, while the EMI SE value of 17.07 dB was achieved with undoped MoS_2_/rGO [95]. By altering the kinds of metal cations, M^2+^/M^3+^ molar ratios, and interlayer compensatory anions, a wide variety of particular host topologies and nanostructures with customizable chemical and physical characteristics can be made. To produce EMI shielding of this kind, multilayer double hydroxides (LDHs) are introduced to improve the SE performance of shielding materials. 

### 4.4. Metal-Layered Double Hydroxides

**Figure 12:**
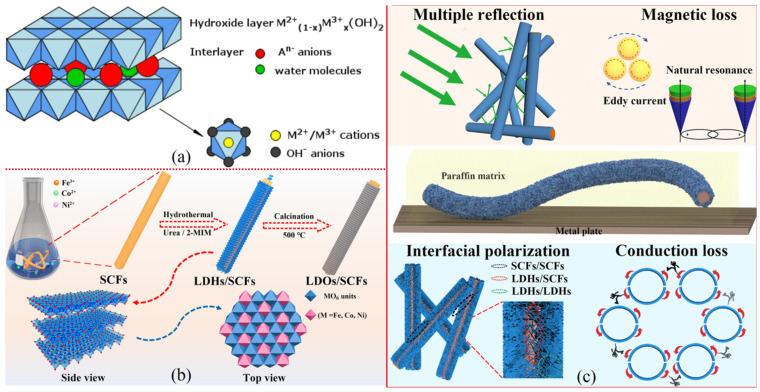
(**a**) Structure of layered double hydroxide [98]. (**b**) Synthesis process, and (**c**) a demonstration of the EMW absorption system of LDHs/SCFs composites [99]. “Reprinted from [99], Copyright (2020), with permission from Elsevier”.

Anionic clays in the class of layered double hydroxides have a lattice resembling a sandwich, wherein the positively charged metal layers sandwich the negative anions in a repeating manner (with generic sequence [AcB Z AcB]_n_; where c, A, and B and Z denote the layers of metal cations, hydroxide anions, and other anions and neutral molecules (such as H_2_O), respectively. The layered structure has good dielectric properties and a wide chemical versatility, making LDHs suitable for EM shielding via dielectric losses [96,97]. Different divalent metals like Cd^2+^, Mn^2+^, Fe^2+^, and Pb^2+^ and trivalent metals like Al^3+^, Cr^3+^, and Fe^3+^ are mixed to produce LDHs. The general formula for the chemical composition of LDHs is $M_{1-x}^{2+}M_{x}^{3+}{\left(OH\right)}_{2}\cdot A_{x/n}^{n-}{\cdot mH}_{2}O$; here, M^2+^ are the divalent and M^3+^ are the trivalent metal ions, A represents interlayer anion, and x and m are fraction constants. A pictorial structure is shown in Figure 12a [98]. LDHs are synthesized with a combination of different divalent like ^+^ and trivalent like ^+^ metals.

Due to their layered structure, LDHs have satisfactory dielectric properties which can be utilize for EM wave attenuation purposes. The publications on the EM shielding characteristics of LDHs are comparatively less than with other materials and not much work has been done on these materials towards an EMI shielding approach. Recently, Z. Zhao et al. prepared an LDH structure specimen as a highly efficient EM wave absorber material. Three transition-metals (Fe, Co, and Ni) were combined into three different layered double hydroxides, FeNi-LDHs, CoNi-LDHs, and FeCo-LDHs, via the hydrothermal method. Thereafter, they were produced on the surface of short carbon fibers (SCFs) using in situ followed by calcination treatment of CoNi-LDHs/SCFs, FeNi-LDHs/SCFs, and FeCo-LDHs/SCFs at 500 °C to improve the EMW absorption, and their calcined products were named as CN-500, FN-500, and FC-500, respectively [49]. The R_L_ values for FN, CN, and FC are 42.9 dB, 13.3 dB, and 29.9 dB with an effectual bandwidth of absorption of 1.68 GHz, 3.68 GHz, and 4.48 GHz, respectively, achieved with a sheet thickness of 2.2 mm for both CN and FN, and the thickness of the FC sheet was taken as 2.0 mm [49]. Figure 12b outlines the absorption mechanisms that lead to the superior EMW absorption characteristics of the composite. The enhanced shielding characteristics are due to the occurrence of natural resonance, eddy current loss, strong interfacial polarization, impedance matching, and electron hopping across adjacent carbon fibers.

Using metal-ligand absorbance, Quan et al. created graphene oxide laminated CoFeAl-LDH hybrids. The thickness of the hybrid composites and its loading amount were changed to improve their MWA performance. In CoFeAl-LDH/GO 50 wt% greatest R_L_ and bandwidth of −23.8 dB and 7.4 GHz were achieved via the Maxell–Wagner–Sillars effect and GO supplied electron driven dipolar polarization at a thickness of 2.5 mm [99]. A 20 mL metal salt mixture of Ni(NO_3_)_2_∙6H_2_O and Fe(NO_3_)_3_∙9H_2_O with constant stirring is added to the ammonia salt and the prepared suspension was annealed for 18 h at 65 °C, resulting in the generation of calcinated NiFe-LDH. The obtained NiFe-LDH has a lower dielectric constant of 3.6 compared to other NiFe-based materials. This material has a reflection loss of −58.8 dB, indicating a good absorption of the incident electromagnetic radiation [100]. A mixture of 1.5 mmol Fe (NO_3_)_3_·9H_2_O, 3 mmol C_6_H_12_O_6_, and 3 mmol Ni (NO_3_)_2_·6H_2_O was prepared and added to 50 m DI water after urea, stirred for 1 h, and, thereafter, heated for 12 h at a temperature of 180 °C, resulting in the formation of FeNi@C microspheres. At a thickness of 1.4 mm, the obtained microsphere has a bandwidth and reflective loss of 4.6 GHz and −10 dB, respectively. The flower-like structure in 3D of the FeNi@C microspheres has a great potential for absorbing the electromagnetic radiations [101]. 

### 4.5. Black Phosphorus

Black phosphorus is an allotrope of phosphorus. In 1914, Percy Bridgman noticed a phase change while examining the impact of high pressure on white phosphorus. He named this new allotrope black phosphorus [102]. This new allotrope was thermodynamically more stable under normal conditions than white and red phosphorus, and its semiconducting characteristics make it a promising material for shielding applications [50,52,103,104,105]. Black phosphorus has a 2D orthorhombic C puckered honeycomb structure with sp^3^ hybridization. It has a tunable thickness-related band gap having range between 0.3–1.5 eV with high charge carrier mobility of approx. 1000 cm^2^V^−1^S^−1^. As far as we are aware, there are not many reports on EMI shielding techniques for BP-based composites. [50,51,52]. The challenges with BP’s breakdown and synthesis make it difficult to work with. Wu et al. fabricated few-layer black phosphorus (FL-BP) nano-sheets using the liquid phase exfoliation (LPE) method followed by mixing 30 and 50 wt% of FL-BP evenly mixed with wax at 85 °C. With 2.5 mm thickness and 30 wt% filler loading of FL-BP in composites, up to 6.20 GHz, the bandwidth (R_L_ < 10 dB) was attained [50]. In another study, Hao et al. produced an extremely light rGO/BP composite via aerogel as a lightweight microwave absorber material using a facile self-assembled technique. The rGO 3D framework had BP nanosheets dispersed uniformly across it, creating a high porosity with superior shielding capabilities. For rGO/BP composite aerogel with a 2.53 mm thickness, an R_L_ of 46 dB and broad effective absorbing bandwidth of 6.1 GHz (R_L_ < 10 dB) were achieved [51]. Later on, Zhao et al. fabricated highly durable BP-graphene sheets that demonstrated environmental stability, high mechanical toughness, and highest-ever-recorded toughness (∼51.8 M·J·m^−3^) using GO and BP hybrid films. The BP content increased the electrical conductivity from 8.5 ± 0.6 up to 22.7 S·cm^−1^ and the films made of graphene showed exceptional EMI shielding abilities. For BP-graphene of thickness 5 µm at 8 GHz, EMI SE of 29.7 dB is present, which is twice the EMI SE value of the initial rGO sheet (14.8 dB). The layered structure and joint actions of the rGO sheets and BP were credited with the enhancement in EMI SE values [52].

### 4.6. MXenes

**Figure 13:**
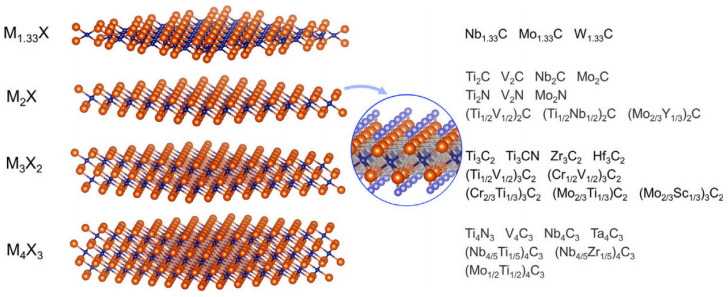
Structure of MXene obtained so far. “Reprinted from [108], Copyright (2019), with permission from Elsevier”.

MXenes fall under the category of 2D inorganic compounds that contain layers of transition metal carbides, carbonitrides, and nitrides that are only a few atoms thick. Mono-M elements, ordered out of plane double-M elements, ordered in plane double-M elements, solid solutions, vacancy ordered, and vacancy randomly distributed are the six potential structures in MXenes. A class of layered ternary carbides and nitrides with the formula $M_{n+1}AX_{n}$ (where n = 1, 2, or 3, A is a 13 or 14 group element of periodic table, and X is carbon and/or nitrogen) and M represents the transition element, are known as MAX phases. Gogotsi et al. first described MAX phases in 2011, and, upon etching the A elements or removing the intermediate element from MAX phases, the result is the MXene [106,107]. The general formula of MXenes is $M_{n+1}X_{n}T_{x}$, where T_x_ represents a vanishing surficial functional group (OH, O/F), schematically shown with via Figure 13 [108]. 

MXenes are hydrophilic and have metallic electrical conductivity (due to their OH or oxygen vanished surfaces). Due to their high stability and exceptional electrical and heat conductivities, MXenes are among the several 2D materials that are being investigated for use in EM wave attenuation. For EM waves to reflect in the shield’s core, MXenes and their composites with their layer-by-layer porous and segmented morphology provide additional interfaces (or internal scattering). The process of internal scattering increases the likelihood that EM waves will contact with the shield and causes more EM waves to transit inside the shielding material before transmission, leading to greater attenuation of EM waves through absorption [109,110]. An increase in absorption loss is brought on by internal scattering, which is created by additional interfacial surfaces inside the shield, but the number of internal reflections that occur between the surfaces of the shield decreases the effectiveness of shielding.

Research on MXene for EMI shielding applications has grown rapidly following the first work on EMI shielding performance of MXenes investigated by Gogotsi and coworkers in 2016 [53]. The majority of the literature reported Ti-based MXenes owing to their ease of accessibility. Flexible free-standing MXene sheets (Ti_3_C_2_T_x_, Mo_2_TiC_2_T_x_, and Mo_2_Ti_2_C_3_T_x_) are prepared using colloidal solutions of MXenes with vacuum-assisted filtration. Due to its electrical conductivity of 4600 S·cm^−1^ among other samples and multiple internal reflections (MIRs) from Ti_3_C_2_T_x_ flakes in the free-standing films, a 45 µm-thick pure Ti_3_C_2_T_x_ film demonstrated EMI shielding performance of 92 dB. The layered structure of MXenes obscures several internal reflections, which are important and are combined with absorption when re-reflected waves dissipate as heat inside the substance. Due to the metallic nature, pristine Ti_3_C_2_T_x_ MXene film displayed better electrical conductivity. Conversely, the lowest value of electrical conductivity (119.7 S·cm^−1^) was noted for Mo_2_TiC_2_T_x_ as being like a semiconductor. The EMI SE value of the Ti_3_C_2_T_x_ film in the X-band was nearly 50 dB at a thickness of 2.5 m [53]. 

**Figure 14:**
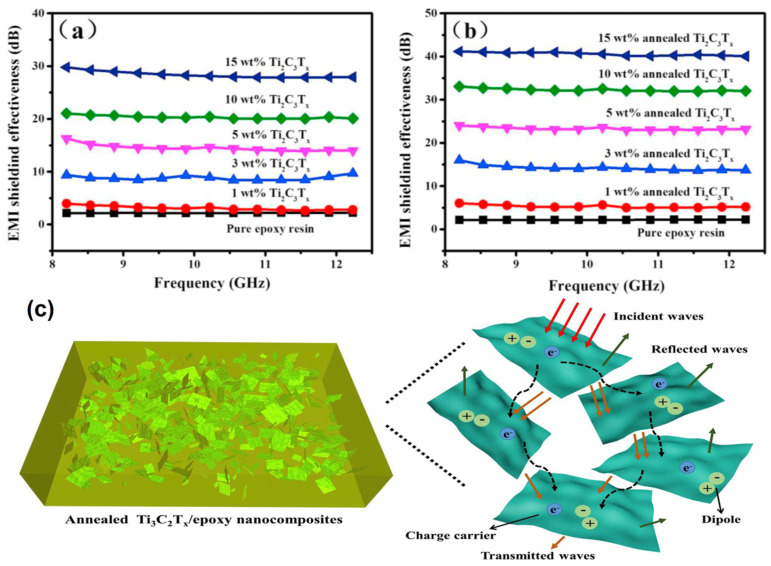
Shielding effectiveness of (**a**) Ti_3_C_2_T_x_/epoxy and (**b**) annealed Ti_3_C_2_T_x_/epoxy samples. (**c**) Schematic picturization of the composites structure and shielding mechanism. Elsevier’s permission is obtained in this reprint. “Reprinted from [111], Copyright (2019), with permission from Elsevier”.

In this line, Shahzad et al. created a Ti_3_C_2_T_x_/SA (sodium alginate) polymer composite with an EMI SE of 57 dB in an 8 m-thick film with 10 wt% SA filler loading [53]. Ti_3_C_2_T_x_/epoxy composites were created by Wang et al. using in situ synthesis centered on a precursor and mixing MXene solution [111]. It was discovered that annealing MXene/Epoxy (EP) composites considerably enhanced their electrical conductivity and EMI SE. Figure 14 illustrates the annealed Ti_3_C_2_T_x_/EP composite’s 41 dB EMI SE in the X-band after loading it with 15 wt% MXene. Because of the impedance mismatch at the contacts, which causes EM waves to be reflected and absorbed by interacting with charge carriers, absorption was the predominant EMI shielding mechanism in MXene/EP composites. More interfaces increase the path length of travel of EM waves inside the annealed MXene/EP composites, which results in increased multiple internal reflection and reabsorption by interaction with dipoles. The annealed Ti_3_C_2_T_x_/EP composites showed higher SE_A_ and SE_R_ attributed to the stronger electron transport capability and large number of dipole formations.

**Figure 15:**
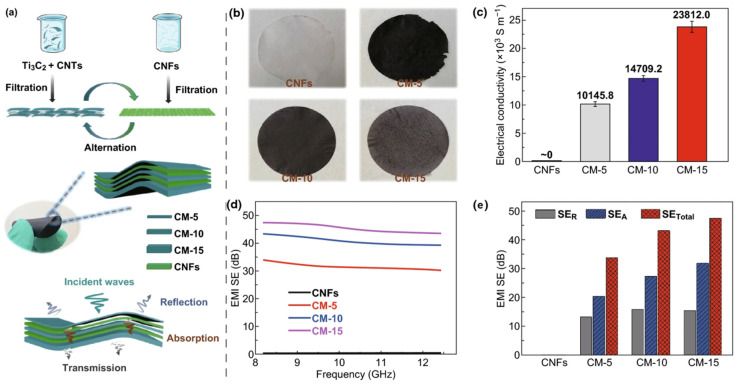
(**a**) The creation of a composite paper’s schematic. (**b**) Digital images, (**c**) representation of electrical conductivity, and (**d**) X-band EMI shielding performance of CNFs, CM-5, CM-10, and CM-15 samples. (**e**) Comparative analysis of shielding results of the samples at 8.2 GHz [114].

Furthermore, with only 0.15 weight percent MXene loading, Xu et al. produced MXene/poly (vinyl alcohol) foam and attained an EMI SE of 28 dB [112]. By in situ polymerizing aniline on the top and interior layers of Ti_3_C_2_T_x_ MXene, Wei et al. created MXene/polyaniline composites for the attenuation of EM radiation. With a tunable thickness range of 1.5–2.6 mm, Ti_3_C_2_T_x_/PANI composites mixed in paraffin medium were able to attain an R_L_ value of 56.30 dB at 13.80 GHz and an effectual absorption frequency of 8–18 GHz (from X-band to Ku-band) [113]. By using a simple alternating vacuum-assisted filtering technique, Cao et al. created an atomically thin, versatile, Ti_3_C_2_T_x_ MXene/cellulose nanofibrils composite paper (CMC GS) with a steep and sandwich composition [114]. CMC GS composite sheet achieved an outstanding electrical conductivity of 2506.6 S·m^−1^ and also an EMI SE of 38.4 dB as shown in Figure 15. The weight ratios of CNTs-MXene (where the mass of CNTs is 1 mg) are taken at 1:10, 1:5, and 1:15, and the single-layered composite sheets thereby formed are named CM-10, CM-5, and CM-15, respectively. Jin et al. fabricated flame-retardant poly (vinyl alcohol)/TMC (PVA/MXene) sheets using a multilayer casting process. The electrical conductivity of a 27 µm-thick PVA/MXene multilayered film with a loading of 19.5 wt% MXene was 716 S·m^−1^, the high thermal conductivity was 4.57 W m^−1^·K^−1^, and the highest EMI SE was 44.4 dB [115].

**Figure 16:**
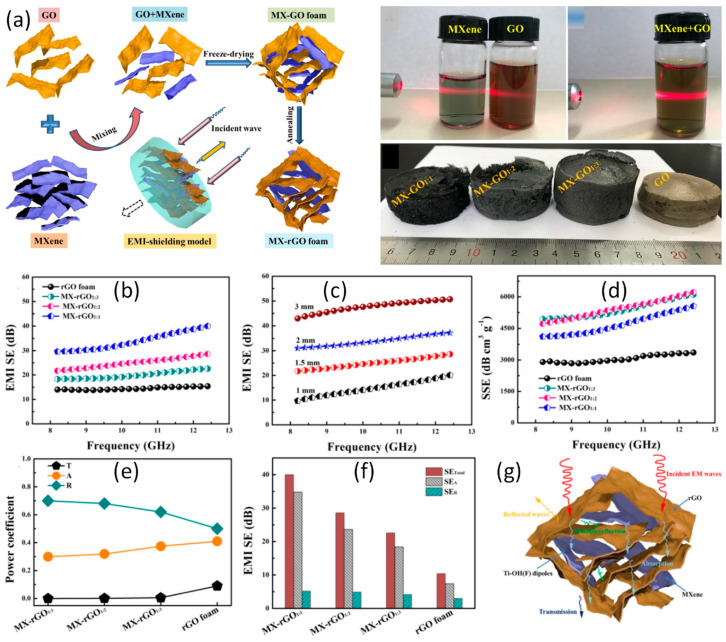
(**a**) MXene/rGO form sample synthesis illustrated schematically with a photograph of the finished product. Shielding effectiveness of (**b**) rGO and MX-rGO composites that are 1.5 mm thick, and (**c**) MX-rGO (1:2) with different thickness. (**d**) Samples’ SSE with a 1.5 mm thickness. (**e**) Values of A, R, and T coefficients of the samples at 12.4 GHz. (**f**) A comparison of the efficacy of shielding caused by reflection versus absorption. (**g**) Depicts the phenomena that happened at the time of interaction of electromagnetic waves with the samples. “Reprinted from [116], Copyright (2019), with permission from Elsevier”.

Moreover, Bian et al. fabricated ultralight MXene aerogel having a density of less than 10 mg·cm^−3^ by direct freeze casting colloidal solutions of MXene without using external supporters or crosslinking surface groups for the first time [59]. In comparison to other materials used for EMI shielding, such as foam-like materials, a Ti_3_C_2_T_x_ MXene aerogel offered an EMI SE up to 75 dB with an incredibly low reflection up to 1 dB. By first incorporating highly conductive Ti_3_C_2_T_x_ MXene films into GO, followed by the defrost process and minimization thermal treatment, as illustrated in Figure 16, Fan et al. created an ultralight MXene/rGO composite foam. The Ti_3_C_2_T_x_/rGO hybrid foam with Ti_3_C_2_T_x_-to-GO mass ratio of 1:1 reached an EMI SE value of 50.7 dB in the corresponding X-band [116]. The porous structure of the Ti_3_C_2_T_x_/rGO hybrid foam promoted higher electromagnetic wave attenuation. Additionally, the MXene/rGO hybrid foam’s SSE/t value (43,690 dB cm^2^ g^−1^) was extremely high.

**Figure 17:**
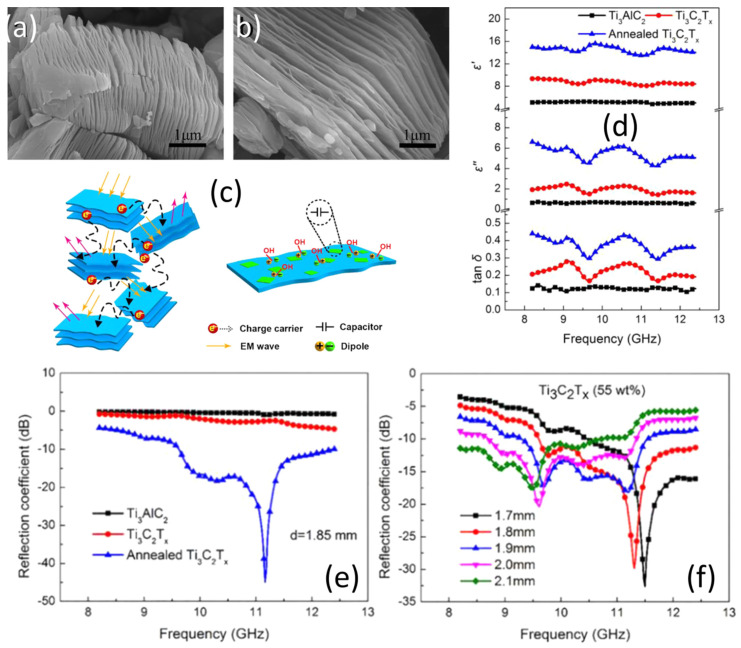
Ti_3_C_2_T_x_ MXene SEM pictures (**a**) pre-annealing and (**b**) post-annealing. (**c**) Diagrammatic representation of the MXene’s absorption of EM wave. (**d**) Permittivity and tangent loss in the dielectric (tan δ) versus frequency for various composites in a wax medium having 50 wt% loading. (**e**) Ti_3_AlC_2_ MAX phase, Ti_3_C_2_T_x_, and annealed Ti_3_C_2_T_x_ reflection coefficient in wax having thickness 1.85 mm, and (**f**) frequency against 55 wt% Ti_3_C_2_T_x_ at various thicknesses. “Reprinted with permission from [54]. Copyright (2016) American Chemical Society”.

Zhang et al. recently developed MXene/rGO porous composite films by utilizing vacuum-assisted filtration and ion-induced self-assembly. With a mass ratio of GO to Ti_3_C_2_T_x_ of 2:2, the Ti_3_C_2_T_x_/rGO composite film has offered a maximum EMI SE of 59 dB. The Ti_3_C_2_T_x_/rGO composite that was created was also more hydrophobic than the Ti_3_C_2_T_x_ film [117]. Han et al. altered the surface of Ti_3_C_2_ MXene made from Ti_3_C_2_T_x_ powders using a quick annealing procedure at 800 °C and 1000 °C for 2 h at a heating rate of 10 °C·s^−1^ in an argon environment. The goal was to improve the EM absorption (EA) and shielding in the X-band. In order to construct electromagnetic wave absorption and shielding composites for a variety of MXenes, it was first necessary to investigate the basic electromagnetic loss process of Ti_3_C_2_T_x_ before and after annealing. According to the microscopic image displayed in Figure 17a,b Ti_3_C_2_T_x_, MXene showed a layered structure comparable to that of pure Ti_3_C_2_T_x_ after being annealed at 800 °C. There was also no discernible TiO_2_ or C phase, resulting in improved permittivity ($\varepsilon_{r}$), dielectric loss, and conductive properties (Figure 17d). In comparison to annealed Ti_3_C_2_T_x_ of comparable thickness, the reflection coefficient values of Ti_3_AlC_2_ and Ti_3_C_2_T_x_ were less than 10 dB, as shown in Figure 17e. Additionally, an annealed Ti_3_C_2_T_x_ MXene film measuring 1.7 mm thick demonstrates a reflection coefficient of 48.4 dB at 11.6 GHz, and a film measuring 1.85 mm thick reaches an effective EA bandwidth of 2.8 GHz, as shown in Figure 17f. Figure 17c depicts a schematic representation of the possible phenomena for EM wave absorption in MXene. The incorporation of TiO_2_ nanoparticles, carbon, and capacitor-like structures favors the interfacial polarization, conductivity, and impedance matching that are the key factors for the enhanced shielding performance [54].

Recently, it was reported that a Ti_3_CNT_x_ MXene film provides more EMI SE than a Ti_3_C_2_T_x_ film. The freestanding films of Ti_3_C_2_T_x_ and Ti_3_CNT_x_ were annealed for 6 h at elevated temperatures in the range of 150–350 °C, and it was found that the porosity of annealed MXene films was increased directly with annealing temperature and provided more interfaces for internal reflections, resulting in enhanced EA. Although the electrical conductivity of annealed Ti_3_CNT_x_ film (1786 S·cm^−1^) is lower than that of the pristine Ti_3_CNT_x_ film, its EMI SE is 116 dB with a 40 µm thickness, which is the highest EMI shielding ever observed for any prepared composite at the same thickness so far [55]. 

MXenes performed exceptionally well at EMI shielding; however, their hydrophilicity made it difficult to use them in moist or wet conditions, which may have affected their reliance and durability. To overcome this environmental issue, Liu et al. used a hydrazine-induced foaming approach to create flexible, autonomous, light, and waterproof Ti_3_C_2_T_x_ MXene foam [118]. The MXene foam exhibited excellent water tolerance and durability due to its unique hydrophobic surface. The size of the foamed MXene films was increased significantly up to 60 µm due to induced pores between MXene layers (which formed an ongoing network by being only partially connected to one another), while the electrical conductivity was decreased on foaming. Although the electrical conductivity of Ti_3_C_2_T_x_ foam was lower (580 S·cm^−1^) than that of its pristine Ti_3_C_2_T_x_ film counterpart (4600 S·cm^−1^), Ti_3_C_2_T_x_ foam provided higher EMI SE (~70 dB) than its pristine MXene film (~53 dB) at the same thickness [118]. It is observed that the SE value is more than 10 dB; it is possible to ignore the contribution of multiple internal reflections. It was observed that SE_A_ increased with increasing thickness of the MXene foam but SE_R_ decreased with increasing thickness, confirming that absorption was the primary EMI-shielding technique in MXene foams, which resulted from the creation of more interfaces for the reflectivity and dispersion of the electromagnetic waves. Another study by Han et al. demonstrated that Ti_3_C_2_T_x_ MXenes in a CO_2_ atmosphere produce layered carbon/TiO_2_ with thicknesses ranging from 1.7 to 5 mm and an effective EA bandwidth of 3.6 to 18 GHz. This material showed a reflection loss of 36 dB [119]. With a matching width of 2.1 mm, Lie et al. created controlled heterogeneous structures of C/TiO_2_ and Ti_2_CT_x_/TiO_2_ nanocomposites from Ti_3_C_2_T_x_ MXene, which offered an R_L_ of 50.3 dB at 7.1 and 14.2 GHz frequencies [120]. Later, 2D titanium carbide (Ti_3_C_2_ MXene) was explored for its shielding applications in the terahertz (THz) frequency range [121]. A colloidal solution of Ti_3_C_2_ MXene flakes is dropped over a nano-slot antenna array, creating a layer that is several tens of nanometers thick. A 500 nm-thick layer on a 500 nm broad antenna was reported to transmit electromagnetic waves less efficiently as frequency increased in the 0.5 to 2 THz range. A boost in the EA properties of the MXene film was guaranteed by the EMI SE values of up to 30 dB at 1 THz [121].

**Figure 18:**
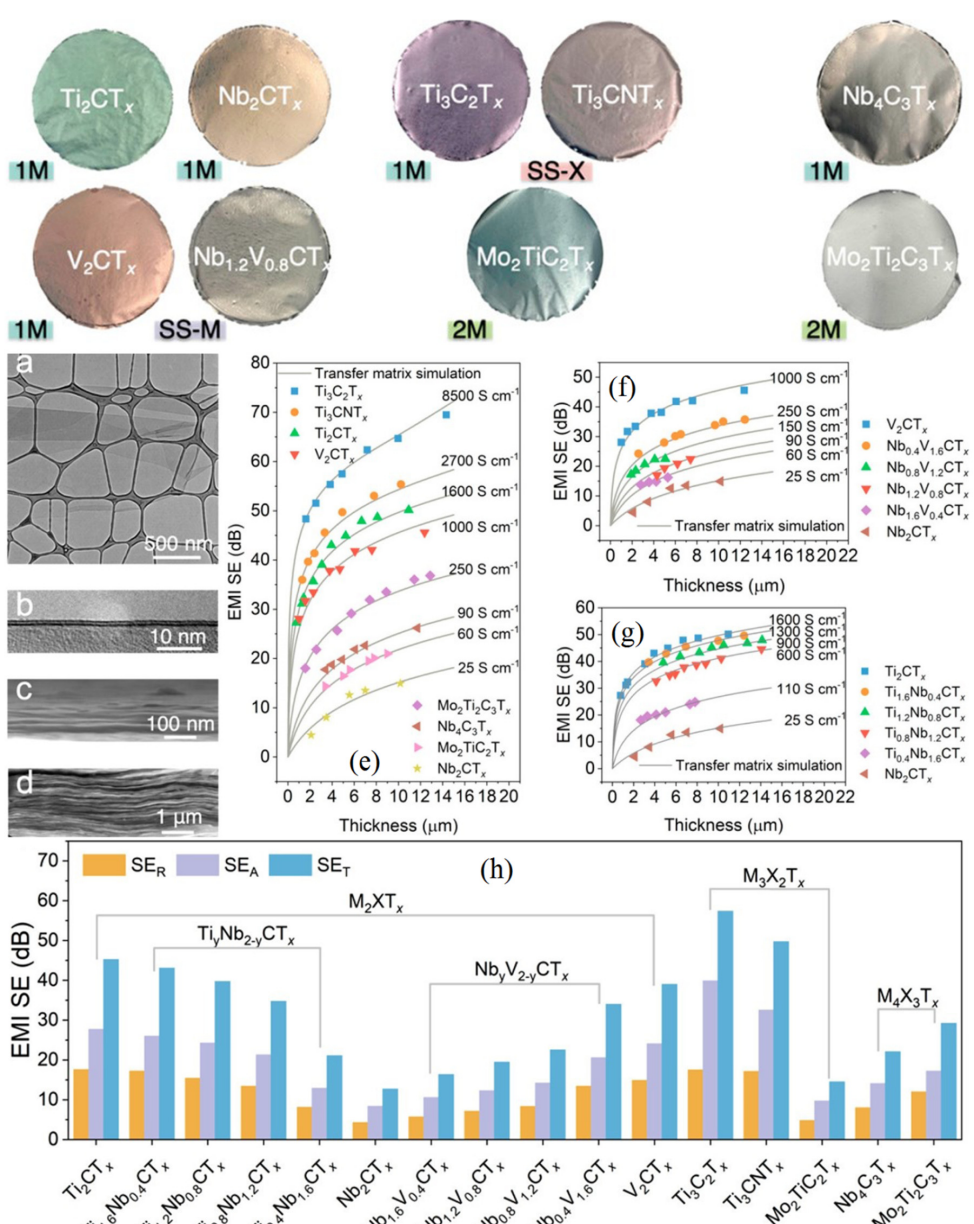
Various films of different MXenes. TEM image of a typical MXene flake (**a**) in-plane view and (**b**) a cross-section of a Ti_2_CT_x_ MXene flake with two layers. (**c**) Cross-section of a Ti_2_CT_x_ film that has been sprayed on a sample holder as seen in a SEM picture and (**d**) vacuum-filtered freestanding Ti_2_CT_x_ film. (**e**) MXene films (M_2_XT_x_, M_3×2_T_x_, and M_4×3_T_x_) with thicknesses ranging from 1 to 15 m at 10 GHz, SE values, and (**f**) EMI SE of Nb_y_V_2−y_CT_x_ (**g**) Different-thickness Ti_y_Nb_2−y_CT MXene films at 10 GHz and (**h**) the typical 8.2–12.4 GHz EMI SE_T_, SE_A_, and SE_R_ of various MXene films, which are each 5 mm thick. “Reprinted with permission from [122]. Copyright (2020) American Chemical Society”.

Figure 18 depicts the results of a comparative study that looked at 16 distinct MXenes (based on Ti, Mo, and Nb) with various compositions and configurations (M_2_XT_x_, M_3×2_T_x_, and M_4×3_T_x_) in order to create EMI-shielding films in addition to Ti_3_C_2_T_x_ [122]. The films were produced using vacuum-assisted filtration, spray-coating, and spin-coating processes, and they displayed a quasilinear characteristic that is dependent on frequency in the X-band. Furthermore, Ti-based MXenes gave maximum SET values (70 dB for 14 µm-thick Ti3C2Tx). Because the chemical process determines electrical conductivity, the solid solution MXenes’ chemistry can be changed to tune the EMI SE [122]. All of the MXene films with a micrometer thickness achieved an efficient EMI insulation of greater than 20 dB. The conclusion of this comparative study was that the EMI SE values for MXenes are tunable with different chemical compositions. 

Terahertz (THz) frequency testing is also used to evaluate Ti_3_C_2_T_x_ ‘s EMI shielding qualities [113,114,115,116]. A systematic density functional theory (DFT) study suggested that the stacked Ti_3_C_2_ flakes and sheets exhibit good THz optical absorption because of the electronic density of states close to the Fermi energy level. Within a bandwidth of 0.25–2 THz, Li et al. conducted an experimental investigation into the charge–carrier behavior of a Ti_3_C_2_T_x_ layer. The considerable absorption that was shown in the layer was explained by the reduction of the THz radiation that was being transmitted. These findings were related to adequate charge carrier density and mobility, which produced outstanding absorbency (46,000 cm^−1^) in the THz band, which lies within the theoretical range, i.e., 450,000–60,000 cm^−1^ [113].

For effective EMI shielding, MXene-based polymer composites and hybrid MXene materials have also been investigated. A 9.17 m-thick composite sheet with a mass ratio of 1:1 of GO to Ti_3_C_2_T_x_ achieved a superior EMI SE with a SE_T_ of 28.2 dB at 12.4 GHz frequency as a result of work by Xiang et al. who generated TiO_2_-Ti_3_C_2_T_x_/GO composite ultra-thin films using vacuum filtering and pyrolysis [61]. To examine how MXene films might be used in portable electronics, a “brick-and-mortar” configuration with a weight ratio of 7:1 of PEDOT:PSS was created by Lie et al. using a vacuum-assisted filtering procedure to create an atomically thin, extensible polymeric Ti_3_C_2_T_x_ sheet. Ti_3_C_2_T_x_/PEDOT: PSS composite material measuring 11.1 m thick displayed a significant EMI SE value of 42.10 dB [57]. In a different work, Nguyen et al. created composites made of Fe_3_O_4_@Ti_3_C_2_T_X_/GF/PDMS that had good EMI SE and also can sense pressure. The composite films demonstrated an average EMI SE of 77 dB in the (Ka-band) frequency range (26.5–40 GHz) and 80 dB in the X-band with frequency range (8.2–12.4 GHz), which is also very helpful for 5G cellular network technology [123]. Using a solution casting method, an ultrathin film of poly (vinylidene fluoride) (PVDF), MXene, and silver nanowires was fabricated possessing electrical conductivity of 1.08 S/m, thermal conductivity of 0.78 W/(mK), and EMI SE 25.87 dB at 300 µm thickness. As the thickness of the film increased from 150 to 600 µm, the value of SE increased from 19.31 to 41.26 dB. The main attribution of shielding is due to absorption of the EM radiation [124].

Ti_3_C_2_T_x_ is injected into Ti_3_C_2_T_x_ MXene/PCF (MPCF) composites to create lightweight, robust, and adaptable materials. An 8.5 weight percent Ti_3_C_2_T_x_ MXene has a 75 dB EMI SE and 216.9 dB cm^3^/g of specific shielding effectiveness. The produced composites have an excellent heat-insulating and flame-resistant performance. In the fields of electronics and electrical engineering, aerospace engineering, and military engineering, these composites offer a variety of possible applications for EMI shielding equipment [125]. For an outstanding EMI shielding and electro/thermal energy storage, a biomass/MXene-based composite is fabricated. In the frequency range of 8.2–12.4 GHz, the EMI SE for the resulting composite is 45 dB. These are multifunctional composites exhibiting wide applications in high-power electronic devices [126]. The main disadvantage of this composite is the total display of the surface functional groups of the MXenes. When handled at high temperatures and with reducible gases, the functional groups present on the surface are eliminated, and the MXenes are oxidized.

## 5. Conclusions and Future Aspects

The rapid advancement of EMI shielding technology studies, the rise in popularity of electronic devices, the issues with EMI contamination, and the possible health dangers for humans and other living creatures make this a topic that is relevant to society. The difficulties associated with EMI have been addressed by a variety of materials, but 2D materials stand out because of their special electrical conductive qualities, high area of the surface, poor density, and unique dielectric properties. This review provides in-depth information on the advancement in the shielding materials and performance of graphene, MXene, nitrides, transition metal dichalcogenides, metal-layered double hydroxides, and black phosphorus, considering their impedance-matching performance, and electric and magnetic losses. Due of its distinctive qualities, including great strength, flexibility, and excellent electrical conductivity, MXene is in the spotlight. In addition to its excellent electrical conductivity, MXene’s diverse chemical make-up, adaptability to manipulation in thin films, and many atom-layers of lattice thickness give it the potential to tune EM energy dissipation paths.

The needs for electromagnetic shielding materials are, nevertheless, increasing as science and technology advance. To satisfy the performance requirements, materials that are flexible, have a broad bandwidth and corrosion resistance, are low-cost, environmentally friendly, and easy to fabricate are required. Among these requirements, the synergy of the proficient attenuation constant and being eco-friendly with thermostability are still challenging to achieve. Research work has been carried out by taking into consideration these properties individually, but there is a lack of knowledge on the integrated design of the structure of the materials and their EMI SE performance. Exploring low-cost, simple-to-operate, and controlled mass production preparation techniques is still required.

The practical implementations are the primary focus of the upcoming research work. To ensure that materials are used regularly, mechanical properties are crucial. Although several studies have recently focused on strength, however, the majority of films that have been produced still exhibit issues with deformation and poor stiffness. Therefore, it is crucial that we employ the extensibility of composites as a primary criterion in order to ensure the appropriate mechanical strength in the succeeding research. The EM shielding materials may respond appropriately when stimulated by changes in the electric field, humidity, temperature, and other environmental factors, which unquestionably broadens their range of applications. It would be interesting to look into how different compositions affect electrical conductivity and, in turn, how effective EMI shielding is. It is crucial to take into account the diversity of the MXenes family as well as how various substrates attach and bond depending on the production technique and the effect of the MXene interface layoff. One of the main future research objectives is the production of MXenes lacking surface functional groups to prevent the oxidation of the MXenes.

## Figures and Tables

**Table 1:** Performance of two-dimensional materials for EMI shielding.

Materials	Loading	Thickness (mm)	Frequency (GHz)	EMI SE (dB)	Ref.
Flexible Graphite	Bulk	3.1	1–2	130	[18]
Carbon foam	20 wt%	0.8	8.2–12.4	24	[19]
Ultrathin carbon foam	Massive	0.024	8–12	24	[20]
Monolayer graphene	Massive	0.34	2.2–7	2.27	[21]
Graphene paper	Massive	0.3	8.2–12.4	46.3	[22]
Graphene paper	Massive	0.05	8–12	60	[23]
Multilayer graphene sheet	Massive	0.018	0.1–18	55	[24]
GN/PET film	__	1.50	18–26.5	55.11	[25]
GO/CNT-Fe_3_O_4_	67 wt%	2.0	12	−19.74 (R_L_)	[26]
rGO film	Bulk	0.0084	8–12	19.1	[27]
rGO/wax	20 wt%	2.0	2–18	29	[28]
rGO/PS composite	7wt%	2.5	8.2–12.4	45.1	[29]
rGO/PS foam	30 wt%	2.5	8.2–12.4	30	[30]
rGO/epoxy	15 wt%	2.0	8.2–12.4	21	[31]
Graphene aerogel film	Bulk	1.4	0.1–3	135	[32]
Fe_3_O_4_/GN	Bulk	0.3 mm	8.2–12.4	21	[33]
rGO/PEI foam	5 wt%	2.5	8.2–12.4	17.8	[34]
rGO/WPU	7.7 wt%	2.0	8.2–12.4	32	[35]
rGO/phenolic	70 wt%	0.3	8.2–12.4	43.4	[36]
rGO/NBR	10 wt%	3.0	7.5–12	57	[37]
Multilayer f-GO/polymer	35 wt%	0.235	8.2–12.4	37.92	[38]
Fe_3_O_4_/GN/PDMS	Bulk	1.0	8.2–12.4	32.4	[39]
Gr-PANI@PI	40 wt%	0.04	8.2–12.4	21.3	[40]
rGO/h-BN	25 wt%	1.6	15.3	−40.5 (R_L_)	[41]
rGO/MnO	Bulk	2.0	11.5–16.4	−38.9 (R_L_)	[42]
GN/CN	10 wt%	1.5	12.8–18	−29.6 (R_L_)	[43]
Carbon@Fe@Fe_3_O_4_	Bulk	1.5	8.6–13.8	−40 (R_L_)	[44]
GF/PANI nanorods	Bulk	2	13.8	−52.5 (R_L_)	[45]
MoS_2_-NS/wax	60 wt%	2.4	9.6–13.76	−38.42 (R_L_)	[46]
rGO/MoS_2_/wax	20 wt%	2.5	13.48	−60 (R_L_)	[47]
MoSe_2_/paraffin	80 wt%	2.56	8.48	−60.23 (R_L_)	[48]
WSe_2_-CIP/paraffin	80 wt%	1.86	12.3	−68.14 (R_L_)	[48]
FeCo-LDHs	Bulk	2.0	12.24–16.72	−29.9 (R_L_)	[49]
FL-BP	30 wt%	2.5	12–18	−46.5 (R_L_)	[50]
rGO/BP aerogel	Bulk	2.53	10	−46.9 (R_L_)	[51]
rGO/BP-AD films	50 wt%	0.005	8	29.7	[52]
Ti_3_C_2_T_x_ free-standing film	Bulk	0.045	8.2–12.4	92	[53]
Ti_3_C_2_T_x/_SA	90 wt%	0.008	8.2–12.4	57	[53]
Ti_3_C_2_T_x_/SA	Bulk	0.011	8.2–12.4	68	[53]
Mo_2_Ti_2_C_3_T_x_ film	Bulk	0.0035	8.2–12.4	26	[53]
Annealed Ti_3_C_2_T_x_	50 wt%	1.7	11.6	−48.4 (R_L_)	[54]
Ti_3_C_2_ MXene/wax	90 wt%	1	8.2–12.4	76.1	[54]
Ti_3_CNT_x_	Bulk	0.04	8.2–12.4	116	[55]
Ti_3_C_2_T_x_CS	Bulk	0.386	8.2–12.4	50.5	[56]
Ti_3_C_2_T_x_/PEDOT: PSS	Bulk	0.007	8.2–12.4	42.5	[57]
Ti_3_C_2_T_x_/PEDOT: PSS	87.5 wt%	0.011	8.2–12.4	42.1	[57]
d-Ti_3_C_2_T_x_/ANF	90 wt%	0.015	8.2–12.4	32.84	[58]
Ti_3_C_2_T_x_ aerogel	Bulk	2.0	8.2–12.4	75	[59]
Calcium alginate/Ti_3_C_2_T_x_	Bulk	3.17	12.4	25.26	[60]
TiO_2_-Ti_3_C_2_T_x_/GO	Bulk	0.0091	12.4	28.2	[61]

## Data Availability

The datasets generated during and/or analyzed during the current study are available from the corresponding author upon reasonable request.
